# Studying Scale-Up and Spread as Social Practice: Theoretical Introduction and Empirical Case Study

**DOI:** 10.2196/jmir.7482

**Published:** 2017-07-07

**Authors:** James Shaw, Sara Shaw, Joseph Wherton, Gemma Hughes, Trisha Greenhalgh

**Affiliations:** ^1^ Women's College Hospital Institute for Health System Solutions and Virtual Care Toronto, ON Canada; ^2^ Nuffield Department of Primary Care Health Sciences University of Oxford Oxford United Kingdom

**Keywords:** sociology, medical, technological innovations, telemedicine, health policy, mHealth

## Abstract

**Background:**

Health and care technologies often succeed on a small scale but fail to achieve widespread use (scale-up) or become routine practice in other settings (spread). One reason for this is under-theorization of the process of scale-up and spread, for which a potentially fruitful theoretical approach is to consider the adoption and use of technologies as social practices.

**Objective:**

This study aimed to use an in-depth case study of assisted living to explore the feasibility and usefulness of a social practice approach to explaining the scale-up of an assisted-living technology across a local system of health and social care.

**Methods:**

This was an individual case study of the implementation of a Global Positioning System (GPS) “geo-fence” for a person living with dementia, nested in a much wider program of ethnographic research and organizational case study of technology implementation across health and social care (Studies in Co-creating Assisted Living Solutions [SCALS] in the United Kingdom). A layered sociological analysis included micro-level data on the index case, meso-level data on the organization, and macro-level data on the wider social, technological, economic, and political context. Data (interviews, ethnographic notes, and documents) were analyzed and synthesized using structuration theory.

**Results:**

A social practice lens enabled the uptake of the GPS technology to be studied in the context of what human actors found salient, meaningful, ethical, legal, materially possible, and professionally or culturally appropriate in particular social situations. Data extracts were used to illustrate three exemplar findings. First, professional practice is (and probably always will be) oriented not to “implementing technologies” but to providing excellent, ethical care to sick and vulnerable individuals. Second, in order to “work,” health and care technologies rely heavily on human relationships and situated knowledge. Third, such technologies do not just need to be adopted by individuals; they need to be incorporated into personal habits and collaborative routines (both lay and professional).

**Conclusions:**

Health and care technologies need to be embedded within sociotechnical networks and made to work through situated knowledge, personal habits, and collaborative routines. A technology that “works” for one individual in a particular set of circumstances is unlikely to work in the same way for another in a different set of circumstances. We recommend the further study of social practices and the application of co-design principles. However, our findings suggest that even if this occurs, the scale-up and spread of many health and care technologies will be neither rapid nor smooth.

## Introduction

### Background

Increasing the uptake of digital health and care technologies is a policy priority around the world. For example, in the United States, the Agency for Healthcare Research and Quality is promoting an ongoing program of research grants intended to rapidly advance the spread and scale-up of health information technologies [1]. In the United Kingdom, a reimbursement program was announced in 2016 to support “medtech innovations” intended to “help cut the hassle experienced by clinicians and innovators in getting uptake and spread across the NHS” [2]. These new policy programs are based on the assumption that if a health technology has been demonstrated as effective and cost saving (sometimes merely on the anticipation of efficacy and efficiency), then its widespread adoption should be supported across the system as a whole. Such initiatives reflect a push to improve the spread and scale-up of service innovations in health and care systems more generally [3-6].

### Theories of Social Practice: An Overview

Attempts to scale up (increase local usage) and spread (extend usage to new localities and settings) health technologies often prove more difficult than anticipated [7,8]. Previous systematic reviews by us [9,10] and others [11-16] have documented the multiple interacting influences that affect the diffusion and spread of innovations (including technologies) in health care and depicted these as operating at multiple levels in a complex system. In earlier field work, we developed a set of methodologies for combining detailed ethnographic studies of the intended technology user with an analysis of the meso (organizational) and macro (eg, policy) context to begin to theorize both the successes and failures of technology implementation efforts [17-23]. These previous studies by our own team, which covered remote booking services, electronic patient records, repeat prescribing systems, personal health organizers, and home-based assisted living, focused on the detailed study of human action in organizational and wider social contexts and prompted us to develop a new theoretical framework that drew on both structuration theory and actor-network theory [23]. Other researchers have used normalization process theory [6,8,24], actor-network theory [25,26], cultural-historical activity theory [27], technological sensemaking [28], technology structuration theory [29], socio-technical systems theory [30], or simply “practice theory” [31,32] to conduct similar studies of technology-related action in a health care context.

**Table 1:** Overview of theories of social practice.

Theory	Overview
(Cultural-historical) activity theory	It focuses on an object of activity, that is, the aim toward which people work collectively to meet an identified need. The notion of an object of activity encapsulates the mutual motivation around which people from different backgrounds come together in the workplace in more or less stable groupings. Knowledge is seen as intimately tied to practice rather than as a “commodity” to be “transferred.”
Sociotechnical systems theory	It proposes that introducing technologies in an organization is a social process that depends on values, mindsets, and engagement. It is also an evolutionary process (sociotechnical systems are grown, not built), hence best achieved by early and active input of front-line workers into the design of redesign of work routines. Sociotechnical systems theory informed early work on human-computer interaction, workplace ergonomics, and human factors engineering.
Structuration theory	It brings together the notion of an external social reality (aspects of context that exist independently of individual actors, such as the economy, the law, and professional codes of conduct) and that of a subjective reality (individuals’ interpretations and perceptions of reality); it views these as reciprocally linked and mutually reinforcing and is centrally interested in the dynamic between structure (external reality) and agency (individual action and judgment).
Actor-network theory	It considers networks of both people and technologies, known as “actor-networks.” They are often highly dynamic and inherently unstable. They can be stabilized to some extent when people, technologies, roles, routines, training, incentives, and so on are aligned. This alignment is achieved (or at least, attempted) through “translation,” which involves the four stages of problematization (defining a problem for which a particular technology is a solution), interessement (getting others to accept this problem-solution), enrolment (defining the key roles and practices in the network), and mobilization (engaging others in fulfilling the roles, undertaking the practices, and linking with others in the network).
Technological sensemaking	It proposes that that technologies introduced into organizations are open to different interpretations. Sense-making—in which members negotiate the meaning of the technology, how it should or might be used in particular contexts, and what benefits and hazards it could bring—is crucial for successful implementation.
Normalization process theory	It depicts the uptake and routinization of technology in health care organizations as generated through four mechanisms: coherence (users coming to understand and make sense of the technology), cognitive participation (users building a community of practice around the use of the technology), collective action (users collaboratively developing and embedding new work routines), and reflexive monitoring (users agreeing on and implementing measures to evaluate program success).

**Table 2:** Origins of, and comparisons between, different theories of social practice.

Theory	Original publication	Country of origin	Disciplinary roots	Emphasis
Activity theory	Leont’ev 1904 (translated 1979) [38]	Russia	Social psychology	Relationship of workers to their shared activity (linked originally to Marxist philosophy of work)
Socio-technical systems theory	Cherns 1976 [36] (updated 1987) [37]	United Kingdom	Social psychology	Design of effective and efficient work processes with goal of non-stressed workers
Habitus and practice theory	Bourdieu 1977 [39]	France	Anthropology and sociology	Theoretical analysis of how human agents’ dispositions and knowledge are reciprocally shaped by external social structures
Post-structural practice theory	Foucault (1979) [40]	France	History and philosophy	Role of discourses and their impact on the body; creation of individual subjects through discursive historical patterns of practice
Structuration theory	Giddens 1984 [41]	United Kingdom	Sociology	Theoretical analysis (drawing on Bourdieu) of the relationship between social structures and human agency
Actor-network theory	Callon & Latour 1986 [42]	France	Philosophy	Explaining how humans and technologies are linked in dynamic and often unstable networks, and what emerges from these networks
Technological sensemaking	Weick 1990 [43]	United States	Organizational sociology	Explaining how workers make sense of technologies in the workplace and negotiate their (changing) meaning as they work to implement them
Technology structuration theory	Barley 1986 [44] and Orlikowski 1992 [45]	United States	Information systems	Explaining the contingency and unpredictability of technology implementation in organizations
Adaptive structuration theory	DeSanctis and Poole 1994 [46]	United States	Organizational sociology	Explaining the contingency and unpredictability of technology implementation in organizations
Contemporary practice theory	Schatzki 1996 [47], Shove 2012 [48]	United States and United Kingdom	Sociology and anthropology	Human experience within fields of practice; interaction between material and social elements of everyday life
Strong structuration theory	Stones 2005 [49]	United Kingdom	Sociology	Detailed empirical methodology for applying Giddens’ structuration theory to study social change
Normalization process theory	May 2006 [50]	United Kingdom	Sociology	Explaining why technologies do or do not become routinized in the workplace
Strong structuration theory adapted for technology	Greenhalgh and Stones 2010 [23]	United Kingdom	Sociology	Explaining technology adoption (and non-adoption) by considering the situated actions of humans within wider sociotechnical networks

All these approaches are, broadly speaking, theories of social practice (Table 1). Whilst their specific emphasis differs (Table 2), they have in common a focus on individual actions and judgments in context. They hold that human agency (ie, what people do) is based on both their general prior knowledge and their situated local judgments about the meaning of particular technologies and particular actions, taking account of the contingent and material features of context. “Context” is differently defined by different scholars [8,10,33,34] but, broadly speaking, it includes both local and more distant social, political, economic, and technical influences, including “scripts” (patterns of how we might be expected to behave), professional and cultural norms (what is viewed as morally correct), as well as laws, regulations, and availability of resources. The purpose of this paper is to provide an overview of theories of social practice and to illustrate their value in understanding efforts to achieve spread and scale of health and care technologies through a single case example of the adoption of a Global Positioning System (GPS) tracking device for people living with dementia.

All theories of social practice view human agency and context as reciprocally interacting and evolving dynamically over time. Different theories have different disciplinary roots, emerged in different countries, and emphasize different aspects of human agency, context, technology, and the dynamic interaction between them (Table 2). There has been much cross-fertilization over the years. For example, the contemporary science of human factors design, which is largely based in the United States and led by engineers [35] draws heavily on earlier work from British organizational psychologists on socio-technical systems theory [36,37].

Despite the differences in theoretical emphasis (Table 2), this empirical work by ourselves and others on technology uptake from a social practice perspective has produced a striking common finding: sustained use of a technology in a healthcare environment appears to be critically dependent on the situated (that is, locally contingent) actions and judgments of technology end-users, and these actions and judgments are in turn directly influenced by local contextual factors and indirectly influenced by more distant ones.

### Theories of Social Practice in Health Care

The actions of human actors in a healthcare setting are not merely “behaviors” that can be analyzed in isolation from the context in which they occur. Rather, these actions have social meaning and (sometimes) moral significance. Furthermore, they are both shaped and constrained by the material affordances of technologies, which are influenced by such things as technical standards and the assumptions that have been built into technologies as scripts (for example the technology designer may assume that a doctor may give an instruction and a nurse carry it out—a naïve and outdated view of inter-professional teamwork but one that would be scripted into the technology) and access privileges (for example, that administrative staff need not have access to the clinical aspects of a patient’s record). Similarly, *inaction* (“resistance” to technology use) may also be socially, morally and even politically significant and/or materially constrained in socially determined ways. In the study reported here, we applied a social practice lens to explore the different kinds of contextualized social practices on which the scale-up and spread of health technologies depends.

An example of a social practice contributing to technology uptake is the different ways in which people interact with their friends and families via social media apps on their mobile devices. This particular practice carries *meanings related to the evolving role of mobile devices* in our everyday lives, only exists within the *context of the growing penetration of mobile devices* in the global population, and *reproduces assumptions and patterns* of appropriate ways to interact with friends and family (and the appropriate use of these of mobile devices in public places). The rapid spread and scale-up of personal use of such technologies is made possible by the affordability of social media apps (many are free to download), their widespread interoperability with existing platforms, and the lack of legal or regulatory barriers.

By contrast, the use of mobile apps by health care professionals in the context of delivering care is heavily constrained by the prevailing legal and regulatory context and may require changes to the professional scope of practice and/or codes of conduct. The question of whether medical apps should be formally appraised, approved, and regulated for safety reasons has been much debated recently [51]. The United States Food and Drug Administration [52], European Commission, [53] and United Kingdom Medicines and Healthcare Devices Regulatory Agency [54] have attempted to do so (with partial success), but the field remains contested and progress in introducing apps into routine clinical practice remains extremely slow [55]. In short, the social practice of using an app *in the context of health care work* is influenced by a host of both local and more distant contextual issues.

The same can be said for other technologies. Empirical studies adopting a social practice perspective have focused largely on the proximal elements of context, that is, the immediate organizational and material factors that shape and constrain practice. However, these approaches have huge potential to also consider more distal and indirect social, technological, political, and regulatory influences on the practices upon which spread and scale-up of digital health and care technologies depend. Many of the identified barriers to technology uptake operate at what might be called the “macro” level of national policy, regulation, the economic value chain, arrangements for contracting and reimbursement, and other system-level structures [7].

It follows that instead of focusing solely or predominantly on individuals and groups within local organizations, researchers might better spend their time trying to understand the dimensions of entire “fields of practice” (eg, entire health systems) in which implementation initiatives are taking place. An empirical example of this is our study of why primary care clinicians “resisted” the introduction of an electronic booking service for outpatient referrals and were not amenable to crude behaviorist incentive schemes: their reluctance was traceable to deep-seated opposition to a policy of introducing overt competition between secondary care providers (which they considered to be at odds with their professional code of conduct) [22]. Arguably, it is these broader fields of practice that are most relevant for the scale-up and (especially) spread of health technologies, though the immediate local context is also often the key.

### Objective and Research Questions

To illustrate how a social practice approach can allow analysis of both proximal and distal contextual influences on spread and scale-up, we describe an example of the implementation of a care technology—a Global Positioning System (GPS) “geo-fence” for people with dementia. Drawing on the real-life case of 76-year-old Rahim (pseudonym)—a Pakistani man living with dementia, we illustrate how a social practice approach can help us understand why and how implementation of GPS technology occurs. Whilst our own chosen analytic approach to this case uses a particular middle-range theory with which we are familiar, we believe that the potential for studies of social practice to reveal distal barriers to spread and scale-up applies to all the theoretical approaches listed in Tables 1 and 2. In other words, we take the view that the commonalities among the numerous approaches to studying technology use as social practice are more important than their differences.

Our research questions were as follows:

1. At an empirical level, and using detailed analysis of a single case, what explains the difficulties with spread and scale-up for a particular technology?
2. At a more abstract level, what kind of insights can a social practice approach provide that will inform the study of spread and scale-up for technological innovations in health and care more generally?

## Methods

### Study Design

This study was part of the Studies in Co-creating Assisted Living Solutions (SCALS) program based at the University of Oxford, United Kingdom, which is following six case studies of health and care organizations as they strive to improve the care of people with multimorbidity in their own homes with the help of assisted-living technologies. A detailed background and methodology for the SCALS program has been published elsewhere [33]; the study builds on previous work that explored the lived experience of assistive technologies using home-based ethnography [19,56], explored the use of co-design methodologies in customizing such technologies with input from industry and care organizations [57], and developed a set of quality standards for telehealth and telecare [58]. SCALS builds on these previous studies by focusing primarily on the “meso” (organizational) level, considering how the organizational roles, routines, and practices required to embed technologies into business as usual are developed and sustained. This meso-level work is supported with an action research component and informed by micro-level ethnographic case studies of individual patients and clients (the intended technology users), focusing in particular on the work practices of organizational members as they interact with these intended users and with one another. It is also informed by macro-level studies of the wider policy context and political-economic influences. Data sources include semistructured interviews, ethnographic field notes, documents (eg, business plans, correspondence, policies, and protocols) and analysis of the material features and affordances of the technologies being introduced.

The six case studies in SCALS are introducing a wide range of technologies in different settings across the United Kingdom from virtual wards to telehealth support and disease-specific self-management programs. Our theoretical perspective has been developed previously and is based mainly on Stones’ empirical extension of Giddens’ structuration theory [49], enhanced for the study of technology use by Greenhalgh and Stones using selected concepts and terminology from actor-network theory [23]. We return to this approach in the Discussion.

The case we present here addressed the implementation of a GPS tracking system intended to help people with cognitive impairment go out for walks unaccompanied. “Wandering” is depicted as one of the most challenging behaviors displayed by people living with dementia [59]. It is defined as involving “a tendency to move about in either a seemingly aimless or disorientated fashion or in pursuit of an indefinable or unobtainable goal” [60]. “Wandering” raises safety concerns, for which a potential technological solution involves the person wearing a GPS tracking device (eg, on a wrist band or belt) that alerts relevant caregivers (often a remote monitoring center in the first instance, who in turn contact a nominated caregiver) when the device leaves a pre-defined geographical area (a previously agreed upon “geo-fence”). GPS is envisaged as a future “scalable” technology for use with a wide range of people living with dementia and related disorders [61]. Yet, in the setting we were studying only 7 clients, where 30 individuals had been provided with the technology (within a larger regional population of approximately 1500 people living with dementia in the local region), and only around half of those who were provided the technology were actually using it.

The implementation of a GPS device to track the movements of a person with dementia might at first seem quite simple: just attach it to the person’s wrist. But this neglects the substantial technological support, expert consultation, and commitment of local caregivers necessary to make the technology “work.” Attaching a tracking device (whether overtly or covertly) to the body of someone whose ability to give informed consent may be impaired is a socially meaningful and morally-laden act [61]. One person’s “safety technology” is another’s infringement of autonomy. The contested social meaning and ethical implications of the GPS device are central, not marginal, to the success of the service.

To illustrate these issues in more detail, we use the example of Rahim, a 76-year old man, originally from Pakistan and currently living in a large city in England with his adult son and his son’s family. Rahim has dementia, is hard of hearing, and does not speak English. His daughter-in-law, Shakila, is the primary caregiver but does not speak much English, either; Rahim’s two granddaughters Bharti and Labani often translate for health and social care providers. The family is eager to help facilitate the use of the GPS technology because Rahim has been leaving the house for long periods of time, becomes aggressive if his family tries to stop him from leaving, and his behavior has been drawing attention from neighbors. Kate, an occupational therapist, is supporting Rahim and his family in introducing the technology.

### Data Collection

To study the organization’s attempts to assure Rahim’s safety and reduce the stress experienced by his family through the implementation of a GPS device, one of us (JW) made a total of three visits to Rahim’s home between October 2015 and March 2016. As well as interviewing Rahim and his family with the aid of an interpreter, JW made extensive ethnographic field notes both contemporaneously and as soon as was practicable after leaving. JW also conducted phone contact with family members between the home visits and three interviews with staff from the care organizations involved, including his occupational therapist and two telecare coordinators responsible for the provision of the technology. The data collection related to the case of Rahim was supported by wider fieldwork within the organization (and collaborators) involved in the provision and use of the GPS tracking technology. This included shadowing and “naturalistic” interviews with health and social care service providers (occupational therapists, telecare coordinators, and commissioners), monitoring center operators, and technology suppliers. Similar ethnographic data was collected on six other index cases. Finally, we made detailed analyses of the different GPS technologies being offered to support people with dementia, focusing on their material properties and affordances in the context of use (or reasons for non-use).

### Analysis

The multi-modal dataset was stored on NVIVO software and converted into interim summaries of individual cases using narrative as a synthesizing device. Each individual case narrative was between four and five pages long and included extensive quotes and annotations; it presented a brief history of the person’s medical details and social situation as well as a longitudinal account of how they came to be offered a GPS device and how their experiences with it unfolded. In all cases, the narrative included several specific situations in which the device was rejected, failed to work as expected, and/or generated unintended consequences. In a second stage of analysis we applied a set of questions to these small-scale social situations, asking (for example) “what assumptions have been built into this technology about who will take what action and how in this situation?” “What professional codes and standards may be driving the behavior of the staff member at this point in the narrative?” and “what does person A assume about person B’s role or perspective?” In this way, we were able to use a sample of small-scale efforts as “troubleshooting” to surface a complex set of interacting influences, both proximal and distal, on the unfolding of social action.

## Results

### Overview

We chose to report Rahim’s case in detail because it illustrates a number of more general findings from the 7 individual cases within the GPS tracking case study. In qualitative research, there is always a trade-off between depth and breadth, and the study of social practices requires in-depth analysis of small-scale social situations. Rahim’s is largely but not entirely a case of “successful” implementation, and hence it offers an opportunity to analyze carefully the unique combination of people, circumstances, and technologies that account for such success. Below, we give three examples of empirical data on the social practices of care staff and family members that illuminate the context in which GPS technology for people with dementia is implemented and in determining the success (or otherwise) of any future scale-up initiative.

### Professional Judgment and Technology Implementation

Our first data excerpt illustrates how professional practice is oriented not to “implementing a technology” but to delivering a personalized care solution that improves the individual’s quality of life as well as safeguards the vulnerable. Sometimes, these two important goals are at odds with one another (it can seem impossible to achieve safety without burdening the individual with constraints), so creativity and compassion are needed to generate individual solutions.

Take the following observational note in which Kate demonstrates her professional reasoning when helping Rahim and his family use the GPS technology. The family had just finished explaining to Kate that Rahim (who used to work as a tailor and enjoys collecting buttons) has been venturing into the street when his family is unaware, and, while wandering, sometimes eating discarded, rotten food:

Kate is serious and concerned and takes out her A4 notebook to jot this all down. She does not think it is safe for him to be attempting to eat rotten food and is further concerned about his risk of falling or being hit by traffic when engaging in this activity. Her attention is drawn to ways of reducing Rahim’s desire to find things on the street...She comes up with an idea for the family to place buttons and other interesting materials around the garden, which Rahim can then search for and collect. This may help occupy Rahim’s time and allow him to do something he enjoys, without the risks associated with leaving the house. Both Shakila and Bharti [granddaughter] appear optimistic about the idea and say they will give it a go.

In this example, Kate manages to find a possible way of providing Rahim with a meaningful activity that could *replace* his outdoor wandering behavior. If this plan were successful, the GPS technology would no longer be necessary in the same way it was when initially introduced to the family and actually might not be needed at all.

The key point here is that Kate’s effort to implement the technology was not driven solely by her desire to see the technology used, but by a more holistic professional assessment of the individual in his family context. When Kate undertook her initial assessment of Rahim for the possible supply of a GPS device, she was influenced by (among other things) the United Kingdom Mental Capacity Act 2005 [62], including the principle of pursuing the least restrictive option when making decisions, and therefore not interfering with Rahim’s freedom of action to leave his home. This legislative framework represents important elements and principles of the routine Kate is performing as she works to implement the technology.

Notwithstanding the policy goal of “scaling up” the GPS technology for the management of wandering, then, it is not only good professional practice but also a legal requirement to view this technology as desirable for *some but not all* individuals exhibiting wandering behavior. This should not be seen as a “barrier to scale-up,” but as the provision of appropriate, family-centered care.

In a previous study of resistance to technology use by clinicians [20], we asked the question “what is excellence in professional practice?” We concluded (page 20) that:

Good clinical practice involves judgement and attention to the particularities of the patient and their situation (the ‘existential patient’) as well as up-to-date knowledge and incorporation of best scientific evidence (the ‘objective patient’). It follows...that technologies which support the latter at the expense of the former are likely to be experienced by clinicians as interfering with excellent care.”

20

In the example above, Kate makes the (unconscious) professional judgment that the GPS tracking device is irrelevant to the optimal solution for Rahim. Indeed, it is likely that Kate would define her professional role as caring for Rahim, *not* implementing technology.

This example affirms previous studies that have emphasized the unpredictability of scale-up efforts [7,9,46], because it depends on case-by-case decisions about the appropriateness and relevance of the technology that are made iteratively as professionals interact with patients and their families. These decisions simply cannot be specified in a linear logic model at the outset of an implementation initiative, but depend on the unfolding of highly individualized contextual factors. More fundamentally, it is unlikely that professional staff will ever accede to the goal of “scaling up” the implementation of a technology at the expense of individual client needs.

### Personal and Professional Relationships

Our second data excerpt illustrates how, in order to “work,” technologies rely heavily on human relationships and situated knowledge. Our ethnographic observations and interviews showed how Rahim’s daughter-in-law Shakila and granddaughter Bharti spent a lot of time learning about and supporting the GPS technology. They had to be able to operate it and ensure that it was charged every night and switched on, and they had to persuade Rahim to put it on before going off on his walks. They also cared deeply about Rahim and had an intimate knowledge of what mattered to him and how he was likely to behave in particular situations. This allowed them to make judgments about what particular alerts from the technology meant in practice, and particularly, whether Rahim was likely to be “safe” or not, given his specific location.

It was also the case that the professionals involved developed a close knowledge of Rahim and his extended family, and provided what is sometimes known as “relationship-based care” to support their use of the GPS tracking device. In other words, the professionals’ knowledge was not limited to how the technology worked in general; they learnt how Rahim and his family were using it, their individual and collective capabilities in relation to the technology, and their interactions with the wider health and care system. Furthermore, Kate had developed professional relationships with others involved in supporting the technology, such as Chris, the senior engineer responsible for troubleshooting technological issues. Decisions and recommendations about how to use the device drew on this knowledge (which was both explicit and tacit) of the circumstances, needs, and perceived obligations of others involved in Rahim’s care. For example, understanding the family’s close contact with Rahim and knowing Chris’ willingness to modify the parameters around the technology for her clients, Kate was able to facilitate the family’s request to de-activate the emergency button that was being inadvertently (accidentally) pushed throughout the day.

To the extent that the GPS technology “worked” for Rahim, it was due to Shakila and Bharti being available to respond whenever they received an alert that Rahim had crossed over the pre-defined geo-fence that had been programmed into the GPS device. Furthermore, in order to respond to the alert about Rahim’s “objective” location (ie, the pinpoint on a map), these family members also factored in a substantial amount of tacit knowledge about what that location meant subjectively (ie, the relevance to Rahim, his everyday life, and memories) and in terms of Rahim’s safety. Bharti explains in the following interview extract:

Like, if he has gone to the corner shop, [the call operator] will say he is out of his boundary, but we know he comes back [from there]. But, if he doesn’t come back within ten minutes, we will look where he is...About three times a week [we get a call], and twice out of that three we know where he is. And then once we don’t know. If they say three roads away or further, we know he is not familiar with the area. Someone will pick him up...Usually we get someone like my sister, auntie, someone who drives and they will be on the phone.”

What Bharti references here are the underlying social networks and arrangements that support the GPS technology; it relies on one or more dedicated caregivers who can be contacted at any time, who own a phone, who can communicate in English, and who have transportation (in this case, a private car) available to search for Rahim. The success of the technology also relies further on a larger group of people within the social network who can help search for him, if necessary.

As noted above, technologies are designed based on particular assumptions about how and by whom those technologies will be used. This is not a criticism of technology designers (it could not be otherwise), but the assumptions made by designers about who will use it and what additional knowledge will be required to interpret its outputs have consequences for whether and how the technology will be used, adapted, or discarded (and hence for how easy it will be to scale up and spread its implementation). The GPS technology is *designed* to rely on stable arrangements and relationships, intimate knowledge of the client, and a high degree of commitment and availability, like those we observed in Rahim’s family.

Rahim had the necessary social relationships to support the use of GPS technology within his family. However, this is not the case for all people with dementia who exhibit wandering behavior. This raises a crucial point when thinking about scaling up technology: instead of relying on background assumptions (eg, about the “typical” family, how they interact with one another, and how they seek out health and social care), we must acknowledge the variable social networks and accountability arrangements in which potential users of the technology are embedded. Only by understanding how a particular technology will or will not fit into the caregiving practices made possible by a particular family or other interpersonal relationships can a technology such as this be scaled up successfully.

### Personal Habits and Collaborative Routines

Our third data extract illustrates the general point that adoption of a new technology by a client requires changes in the *practices* adopted by both professional and lay caregivers, and in particular, co-ordination and stabilization of shared practices and routines. Implementing technologies requires changing what people do. In many instances, it requires that people come to use, on a regular basis, some new piece of technology that they did not use before. However, changing what people do “on a regular basis” (ie, every time a certain routine is enacted) is not a simple task. At an individual level, peoples’ actions are embedded in longstanding habits that are connected to their surroundings in important ways. At an organizational level, routines are what align the work of individuals into collaborative work patterns, thereby improving the efficiency and predictability of shared tasks; they are not easily changed, especially when they interact with other routines [63].

In Rahim’s case, the “simple” GPS tracking technology required coordinated input from a number of people including Kate (the occupational therapist), the staff at the GPS call center, Shakila (Rahim’s daughter-in-law and primary caregiver), Bharti (his granddaughter), other family members, and of course, Rahim. Each of these individuals had some accumulated knowledge and experience, and each made situated judgments based on what information they considered to be salient, meaningful, ethical, legal, and professionally or culturally appropriate. They also, to some extent at least, had to understand where the other people in the network were coming from and what contribution each could and would make to Rahim’s support package.

This GPS device “worked” for this particular client, for example, because the family was sufficiently close-knit and well-organized to orchestrate a search in response to an alert, because they fully accepted their responsibility to contribute to the routine, and because either Bharti or her sister were usually available to translate between the English-speaking call center and members of her limited-English-speaking family when notified that Rahim had breached his geo-fence. Indeed, the routine worked so well in the case of this particular family that it is easy to take its elements for granted.

It is noteworthy that in Rahim’s case, the collaborative family routine for keeping Rahim safe *before* the technology was introduced was considerably more challenging than the one the technology was able to support. Bharti described it thus:

When he didn’t have [the GPS tracker] we were always looking for him. We had five different cars go out. It would be 2 to 3 hours. And we would find him in random places...This happened three times, and we were really worried. We were looking for him in different places, it was really hard.”

Importantly, the “success” of the GPS tracking technology in Rahim’s case is not that the technology, in and of itself, kept Rahim safe but that within the context of an existing pattern of caring, it made the collective task of keeping him safe when wandering considerably easier. However, the same technology introduced into a different family network may not support or enhance existing care routines (for example, if key caregivers are out at work all day) and may actually increase the workload on caregivers as it potentially “empowers” the individual to wander relatively safely and so may require caregivers to search and rescue more often.

## Discussion

### Summary

This paper has presented a key theoretical and methodological argument—that the study of social practices has great potential for informing the study of spread and scale-up (and the common problem of *lack* of spread and scale-up) of health and care technologies. We have illustrated this with a detailed worked example of a single case study of an elderly man and his family who were using a GPS tracking device, more or less successfully, to increase his freedom to safely wander in his neighborhood. Data extracts were used to illustrate three exemplar findings. First, professional practice is (and probably always will be) oriented not to “implementing technologies” but to providing excellent, ethical care. Second, in order to “work,” health and care technologies rely heavily on networks of human relationships and the situated knowledge of individuals. Third, such technologies do not just need to be adopted by individuals; they need to be incorporated into personal habits and collaborative routines (both lay and professional).

### Implications for Scale-Up and Spread of Health and Care Technologies

Following Giddens [41], we conceptualized the people involved in Rahim’s case as “social actors” who were, to a greater or lesser extent, knowledgeable and reflexive. They contemplated their actions by taking account of social structures such as norms (in Giddens’ terminology, “structures of legitimation,” ie, what they saw as reasonable and ethical, such as assumptions about the nature of excellence that underpins professional practice), meaning-systems (“structures of signification”—the symbolic meanings and significance that they attached to people, experiences, and artifacts), and rules and regulations (“structures of domination,” ie, what they saw as following protocol or obeying external authority).

Giddens himself did not offer a theorization of technology as part of structuration theory, but as we and others have shown previously in relation to a range of technologies, social structures are typically inscribed (sometimes unwittingly) in the software or other design features of technologies [21,23,44-46,64]. In other words, technologies *assume* that particular social practices will be being followed in particular ways as the technology is used, and when these inbuilt assumptions clash with the actual social practices of real actors, the technology may not be adopted at all or it will be rapidly abandoned.

The literature on non-adoption of health and care technologies is dominated by behaviorist terminology and by proposed solutions (such as “incentives” or “levers”) that do not engage meaningfully with the social structures described above [20]. Our findings suggest that unless we deepen our understanding of the complex and situated nature of technology use, the policymakers’ vision of rapid scale-up of new technologies will not be realized.

As we demonstrated previously in the empirically derived ARCHIE standards for assisted-living solutions, it is critically important to work *with* professional and lay intended users to co-design solutions that align with what matters to people and that are achievable and sustainable in practice (ARCHIE: technologies should be Anchored in shared understanding, Realistic about illness, Co-creative, Human, Integrated, and Evaluated) [58]. This study reinforces that message but also sounds a note of caution: even when co-design methodologies are used, we may never achieve the policymakers’ vision (implied in the opening paragraph in this paper) of high levels of scale-up and spread of technological innovations for health and care, achieved through “mass customization” and systematic implementation strategies. At the very least, we must build in considerably more flexibility and nuance to the menu of technological options available and also work to maximize flexibility and scope for professional judgment in the service models that support their use.

We believe that this study provides important insights into the study of the scale-up and spread of health and care technologies through a social practice lens. The use of a single in-depth example allowed us to illustrate themes that emerged consistently across a larger sample of cases in the GPS tracking study and which are also evident in other technologies being studied in the SCALS program.

### Limitations

This study has limitations. The examples illustrate the general principle that external social structures of various kinds profoundly influence the situated action of human actors, but we did not have space here to explore any of these examples in close theoretical detail (this will be addressed in a future paper to be published in the social science literature). The use of a single “successful” case example raises the question of what additional insights we would glean from the study of “failures.” Even with the other 6 individual case examples, the overall sample size for the GPS tracking study was small. That study was in a single locality, hence it did not offer scope to study *spread* to new settings (though we have recently added a second GPS tracking site in a new locality and will be addressing this issue through further empirical work).

We have included a range of theories under the banner of theories of social practice. Each addresses the relationship between some understanding of “the collective” (eg, social structure) and the individual’s propensity to act in particular ways (eg, agency). Sorting out these constructs and their relationships are open questions across social science disciplines. We acknowledge the ongoing debates on this but suggest that these theories collectively offer instructive guidance for studies like ours.

### Conclusions

Whilst we have tabled one example of how a social practice lens might be used to research the enormous policy challenge of the scale-up and spread of new technologies, we have not produced a definitive methodology for addressing all aspects of this challenge. The task before us is to draw on the data generated by research, combined with real-world experience, to establish and examine scale-up strategies that balance the needs of context-sensitivity with the realities of producing technologies that have potential for mass application.

To begin what we hope will be a productive discussion, we suggest that future social practice research studies might throw light on which elements of health and care technologies will need to be customized to every individual user (and her or his context) and which can be produced in a more standardized way. There is also scope for using prospective implementation studies to research how health and social care professionals manage the tension between the policy pressure to implement technologies “at scale” and the professional need to provide appropriate personalized solutions, whether technological or not. We invite others to suggest additional applications of social practice approaches to the important questions of spread and scale-up.

## Abbreviations

ARCHIE: technologies should be Anchored in shared understanding, Realistic about illness, Co-creative, Human, Integrated, and Evaluated
GPS: global positioning system
SCALS: studies in co-creating assisted living solutions
